# Transcript co-variance with Nestin in two mouse genetic reference populations identifies *Lef1* as a novel candidate regulator of neural precursor cell proliferation in the adult hippocampus

**DOI:** 10.3389/fnins.2014.00418

**Published:** 2014-12-12

**Authors:** David G. Ashbrook, Anna Delprato, Claudia Grellmann, Marieke Klein, Richard Wetzel, Rupert W. Overall, Alexandra Badea

**Affiliations:** ^1^Computational and Evolutionary Biology, Faculty of Life Sciences, The University of ManchesterManchester, UK; ^2^BioScience ProjectWakefield, MA, USA; ^3^Institute of Cognitive and Integrative Neuroscience, University of Bordeaux and CNRSTalence, France; ^4^Department of Neurology, Max Planck Institute for Human Cognitive and Brain SciencesLeipzig, Germany; ^5^IFB Adiposity Diseases, Leipzig University Medical CenterLeipzig, Germany; ^6^Department of Human Genetics, Donders Institute for Brain, Cognition and Behaviour, Radboud University Medical Center NijmegenNijmegen, Netherlands; ^7^German Center for Neurodegenerative Diseases (DZNE)Dresden, Germany; ^8^CRTD - Center for Regenerative Therapies Dresden, Genomics of Regeneration, Technische Universität DresdenDresden, Germany; ^9^Department of Radiology, Center for In Vivo Microscopy, Duke University Medical CenterDurham, NC, USA

**Keywords:** adult neurogenesis, BXD, CXB, neuroinformatics, recombinant inbred mice, systems genetics, *Lef1*, Wnt pathway

## Abstract

Adult neurogenesis, the lifelong production of new neurons in the adult brain, is under complex genetic control but many of the genes involved remain to be identified. In this study, we have integrated publicly available gene expression data from the BXD and CXB recombinant inbred mouse lines to discover genes co-expressed in the adult hippocampus with Nestin, a common marker of the neural precursor cell population. In addition, we incorporated spatial expression information to restrict candidates to genes with high differential gene expression in the hippocampal dentate gyrus. Incorporating data from curated protein-protein interaction databases revealed interactions between our candidate genes and those already known to be involved in adult neurogenesis. Enrichment analysis suggested a link to the Wnt/β-catenin pathway, known to be involved in adult neurogenesis. In particular, our candidates were enriched in targets of *Lef1*, a modulator of the Wnt pathway. In conclusion, our combination of bioinformatics approaches identified six novel candidate genes involved in adult neurogenesis; *Amer3, Eya3, Mtdh, Nr4a3, Polr2a*, and *Tbkbp1*. Further, we propose a role for *Lef1* transcriptional control in the regulation of adult hippocampal precursor cell proliferation.

## Introduction

In the hippocampal dentate gyrus of many mammalian species, including mice (Kempermann et al., 1997a,b) and humans (Eriksson et al., 1998), there exists a population of neural stem cells that continue to divide and give rise to new granule cell neurons throughout adulthood. The proliferation of these precursor cells is under strong genetic control (Kempermann et al., 1997a, 2006; Kempermann and Gage, 2002); and is modulated by a complex interplay of genetic interactions (Kempermann et al., 2006; Pozniak and Pleasure, 2006; Kempermann, 2011). Although many genes have already been assigned a role in the regulation of proliferation in this system (Overall et al., 2012), many more undoubtedly remain to be identified. In addition, the functional interactions between these genes and their protein products have, in most cases, yet to be established.

Murine genetic reference populations provide excellent tools to address such questions since, firstly, neurogenesis is a well demonstrated phenomenon in mice and, secondly, these sets of strains with fixed, replicable genomes model human genetic complexity and how this influences phenotypes. Recombinant inbred (RI) strain families are genetic reference populations constructed with great experimental control on genotypic variation (Williams et al., 2001), and have been used extensively in quantitative trait loci mapping. Because of their genotypic stability, they provide a platform for phenotype and gene expression data that can be shared between different experimenters, across different time points (Collaborative Cross Consortium, 2012). We identified two such resources for which compatible hippocampal expression data exist (Overall et al., 2009): the BXD RI cross between C57BL/6J and DBA/2J (Taylor, 1978; Taylor et al., 1999; Peirce et al., 2004), and the CXB RI cross between C57BL/6ByJ and BALB/cByJ (Bailey, 1971; Nowakowski, 1984).

We chose to focus on the intermediate filament protein Nestin (Nes), first discovered in neuroepithelial stem cells (Lendahl et al., 1990), and a widely-used marker of the proliferating neural precursor cell population in the adult hippocampal subgranular zone (Reynolds and Weiss, 1992; Yamaguchi et al., 2000). Nestin defines a mixed cell population of proliferating cells, including the type-1 stem cells as well as the type-2a and type-2b transiently amplifying precursor cells (Kempermann et al., 2004). Thus, it would be of great interest to identify related markers which may specify sub-populations to allow more accurate phenotyping of these stages. Also, Nestin, while useful as a marker of precursors, is a structural protein and therefore unlikely to be an upstream modulator of cell fate. It would be of great interest to discover molecules regulating the expression of genes in the precursor cell population. The work presented here has thus examined gene expression profiles correlating with Nestin in two mouse genetic reference panels in order to identify genes potentially regulating proliferation of the neural precursor cell population.

Our results are an example of how a bioinformatics approach, using only information already available in the public domain, can be successfully used to generate novel hypotheses, which can be later tested experimentally at the bench, to help better understand some of the open questions in neurogenomics.

## Methods

### Correlation with expression data

We used GeneNetwork (Chesler et al., 2003, 2005), to gain access to Nestin related traits for BXD and CXB RI families of mice. In the Affymetrix M430v2 microarray platform used (http://www.genenetwork.org/dbdoc/Hippocampus_M430_V2_PDNN_Sept05.html), Nestin is represented by three probesets (1453997_a_at, 1418289_at, 1449022_at). The first principal component of these traits was calculated to create a meta-trait, *Nes*-PC1. This *Nes*-PC1 meta-trait was then correlated (Pearson product-moment correlation coefficient) to all probesets in the two hippocampal expression data sets “BXD Hippocampus Consortium M430v2 (Jun06)” normalized by PDNN (GeneNetwork accession: GN112; Overall et al., 2009) and “CXB Hippocampus Consortium M430v2 (Dec05),” normalized by PDNN (GeneNetwork accession: GN99; Overall et al., 2009). The intersection of all probe sets was calculated, using a Pearson product-moment correlation coefficient (*r*) value greater than 0.8 in both data sets. A correlation coefficient cutoff was used rather than a significance threshold due to the large difference in the number of lines between the two datasets (71 BXD lines, 15 CXB lines). The correlation threshold of 0.8 had a *p*-value of < 1 × 10 ^−16^ in BXD and ~0.00014 in CXB. Use of a significance value threshold would have resulted in many BXD genes being included which had a very small, but still significant, correlation. The threshold of 0.8 was chosen empirically as a strong correlation, with the intention of selecting for genes highly associated with Nestin. We determined the significance of the intersecting genes by permuting the *Nes-*PC1 meta-trait data and re-running the analysis 1000 times.

Statistical tests were done using the free and open source software package R (http://www.r-project.org; R Core Team, 2013).

### Differential gene expression search based on the allen mouse brain atlas

The resulting gene list found to correlate with the *Nes-*PC1 meta-trait for both BXD and CXB families was further constrained based on *in situ* hybridization data registered to a common anatomical atlas, available from the Allen Institute for brain sciences (Lein et al., 2007; Allen Institute for Brain Science, 2014; http://www.brain-map.org). These resources allow one to single out genes enriched in areas of interest that are defined in the anatomical atlas. Using a python script, courtesy of Dr. David Feng, we queried the Allen Brain Atlas database to generate a list of possible candidates by examining the differential gene expression patterns for the dentate gyrus, contrasted against the whole gray matter, thresholding the results at a minimum of 2-fold expression enrichment, a threshold that is comparable with values in the literature (Tusher et al., 2001). This produced a list of genes with enhanced expression in the hippocampal dentate gyrus.

### Enrichment analysis of candidate genes

Our candidate genes were investigated using WebGestalt (http://bioinfo.vanderbilt.edu/webgestalt; Zhang et al., 2005; Wang et al., 2013) for enrichment in GeneOntology (GO), KEGG pathways, wikipathways and transcription factor targets. This allowed us to find commonality between our candidate genes. The whole mouse genome was used as the reference set and the Benjamini and Hochberg (1995) method was used to correct for multiple tests.

The Mammalian Adult Neurogenesis Gene Ontology, MANGO, is a database of genes known to be involved in adult hippocampal neurogenesis (http://mango.adult-neurogenesis.de; Overall et al., 2012). The MANGO API was used to produce a subset of genes which are expressed in type-1, -2a and -2b cells, i.e., Nestin-positive cells (http://mango.adult-neurogenesis.de/xml/annotations?process=expression&cellstage=t1,t2a,t2b&effect=positive&expression=true).

### Predicted and known interactions with genes known to be involved in adult neurogenesis

GeneMANIA (http://genemania.org; Mostafavi et al., 2008; Warde-Farley et al., 2010) was used to investigate known and predicted interactions, including protein and genetic interactions, pathways, co-expression, co-localization and protein domain similarity. Our candidate genes, genes known to be expressed in Nestin positive cells and our candidate regulator *Lef1* were submitted to the website and a summary network created. Default settings were used.

A summary figure for the role of *Lef1* was created using Cytoscape (http://www.cytoscape.org; Saito et al., 2012; Su et al., 2014), incorporating the above transcription factor target and interaction data. Further, IntAct was used to find additional known protein-protein interactions (www.ebi.ac.uk/intact; Orchard et al., 2014).

### Correlation analysis in the hippocampus of RI strains

To support links found through protein-protein interactions and enrichment analysis, correlations were carried out between our candidate genes in the BXD and CXB hippocampus microarray data. The probes identified above via the *Nes*-PC1 meta-trait were correlated against two probes for *Lef1* expression (1445568_at and 1454734_at) using the built in functionality of GeneNetwork.

All online analyses and database queries were verified on 24 October 2014.

## Results

### A common set of genes is associated with hippocampal nestin expression in different genetic reference populations

**Figure 1:**
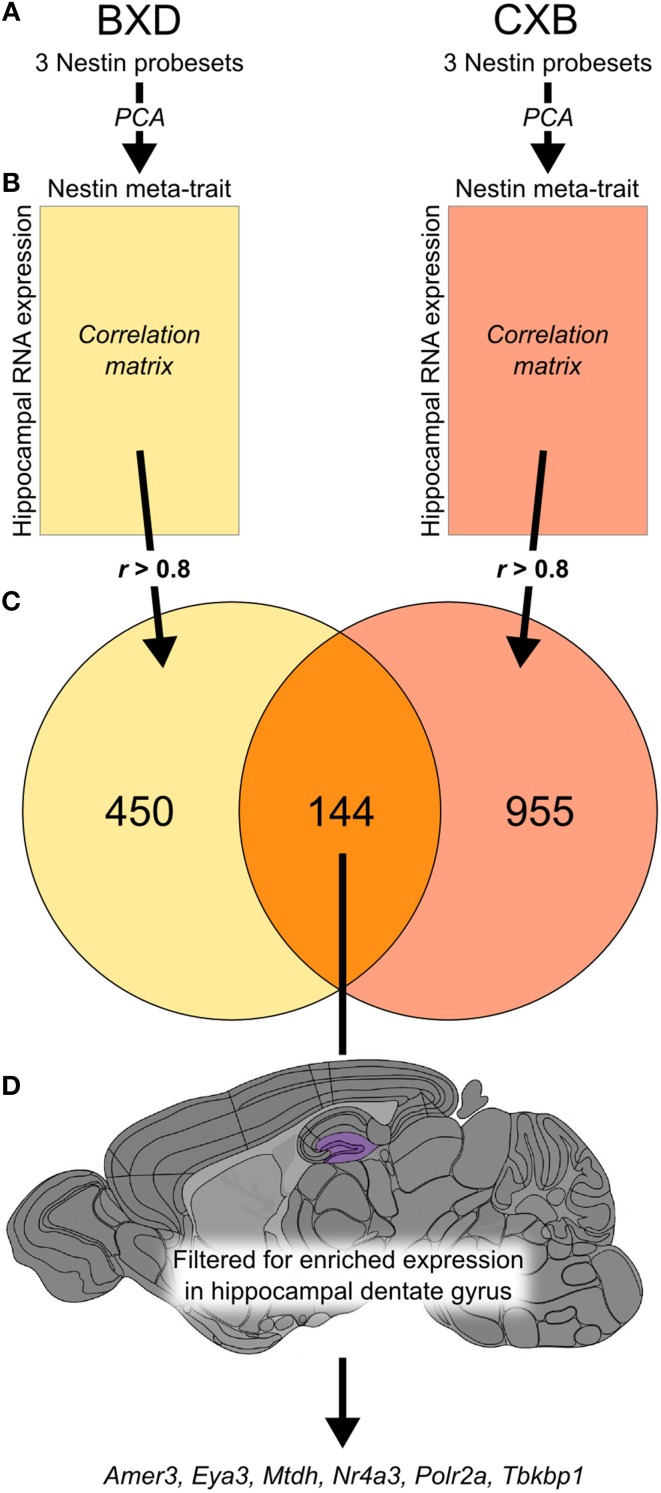
**The integration of data from two genetic reference populations revealed a set of 144 genes potentially expressed in adult hippocampal neural precursor cells**. Principal component analysis was carried out using three probesets for *Nes* expression (1453997_a_at, 1418289_at, 1449022_at) in both the BXD and CXB genetic reference populations **(A)**. The first principal component of these traits was used to create a meta-trait, *Nes*-PC1. This meta-trait was then correlated to all probesets in the two hippocampal expression data sets for the two genetic reference populations **(B)**. The intersection of all probesets (*r*-value greater than 0.8 in both data sets) revealed 144 probesets in common **(C)**. These genes were then filtered based on enriched expression in the dentate gyrus (highlighted in purple) using the Allen Brain Atlas to yield a subset of 6 candidate genes most likely to be expressed in neural precursor cells **(D)**.

Hippocampal expression has been measured previously in two genetic reference populations, BXD and CXB RI strain families, as part of a single experiment (Overall et al., 2009), meaning that the array platform and hybridization handling was common to both. We generated a meta-trait, *Nes*-PC1, based on the first principal component of the expression profiles for the three probesets targeting Nestin (Figure 1A). For each RI population, we correlated *Nes*-PC1 against all probesets on the array and selected strong correlations of *r* > 0.8 (Figure 1B). Of the resulting 450 probesets in BXD and 955 probesets in CXB, 144 probesets (excluding those for *Nes* itself) were common to both sets (Figure 1C; Supplementary Table 1). No overlapping gene sets of 144 or more members were observed after permutation testing of *Nes*-PC1 data, with 1000 such permutations. This indicates that the association of these genes with *Nes* is not due simply to chance.

### A subset of nestin correlates are enriched in the hippocampal neurogenic niche

**Figure 2:**
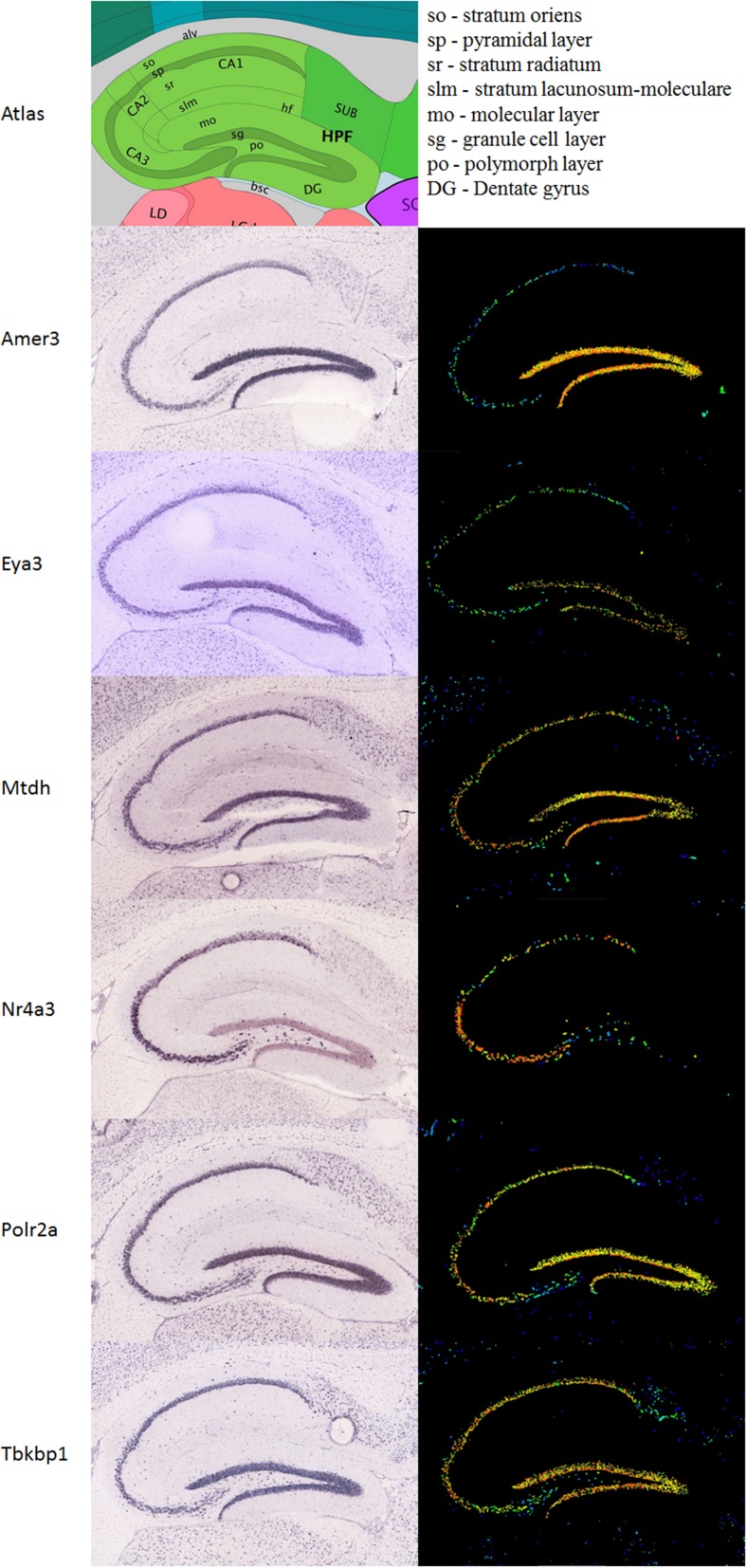
**Expression of the six candidate genes in the hippocampus**. Immunohistochemistry (ISH) is presented in the left column, the expression in the same sagittal slices in the right column. An atlas is shown above to help orientation. All images are taken from the Allen Mouse brain data (Lein et al., 2007; Allen Institute for Brain Science, 2014; http://www.brain-map.org).

A differential search was performed using the Allen Brain Atlas Resource which produced a list of 2472 genes with enhanced expression in the hippocampal dentate gyrus, contrasted against the whole gray matter of the brain. This was used to narrow the list of 144 genes obtained through correlation with our *Nes*-PC1 meta-trait down to six candidate genes (Figure 1D). These candidates, correlating with *Nes* and enriched in the dentate gyrus, are therefore hypothesized to be involved in adult hippocampal neurogenesis. The six candidates are: *Amer3 (Fam123c), Eya3, Mtdh, Nr4a3, Polr2a, and Tbkbp1*. The ISH data and expression intensity for these 6 genes are presented in Figure 2.

### Enrichment analysis suggest lef1 as a common regulator in neural precursor cells

Enrichment analysis was carried out using WebGestalt for our six candidate genes, *Amer3, Eya3, Mtdh, Nr4a3, Polr2a*, and *Tbkbp1*. There is significant enrichment for several GeneOntology terms related to transcription (Supplementary Table 2). For example “transcription, DNA-dependent,” was significantly enriched (Benjamini and Hochberg adjusted *p*-value; adjP = 0.0395) due to *Nr4a3, Eya3, Mtdh*, and *Polr2a*. In addition, the six candidate genes were enriched (adjP = 0.0001) for the transcription factor *Lef1*, which targets *Nr4a3, Eya3, Amer3*, and *Polr2a* (Table 1).

**Table 1:**
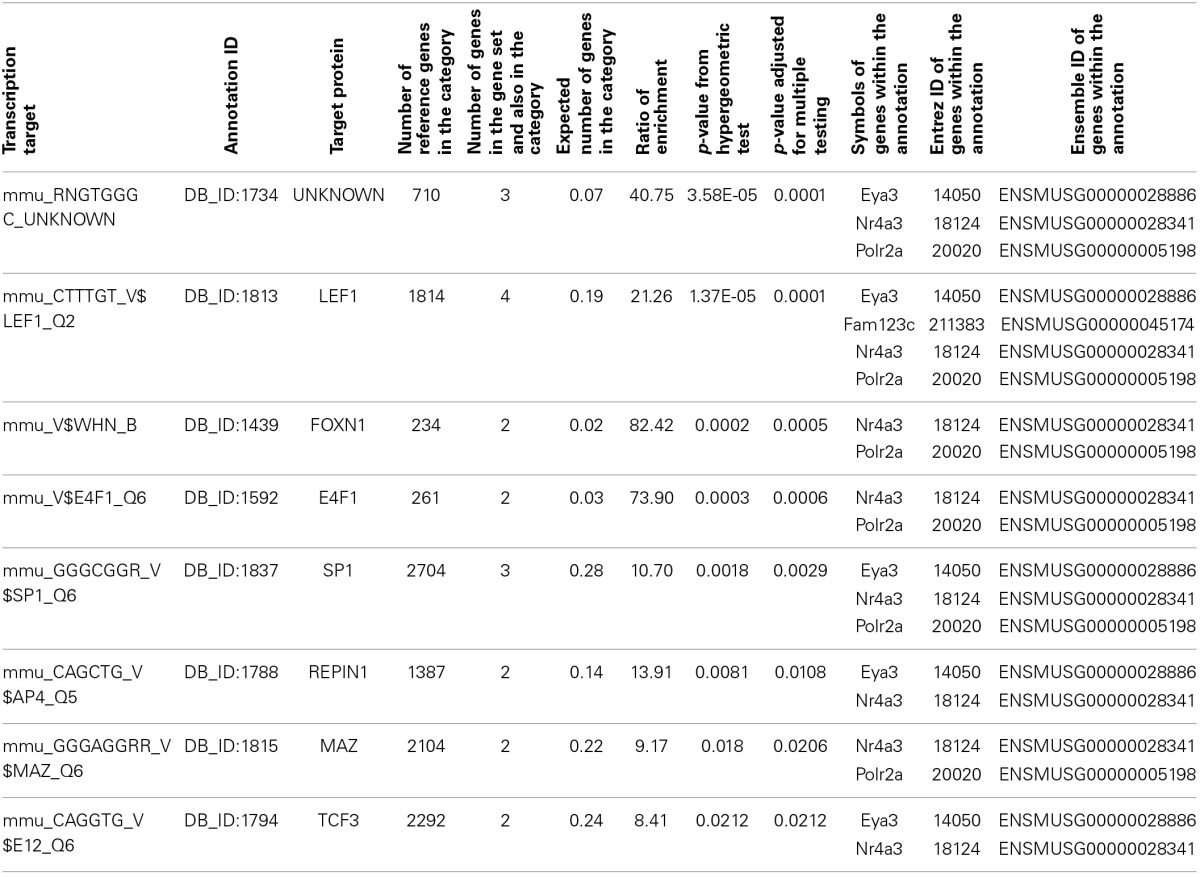
**Transcription factor target annotation enrichment from WebGestalt for our six candidate genes, *Amer3* (*Fam123c*), *Eya3, Mtdh, Nr4a3, Polr2a*, and *Tbkbp1***.

*Showing the transcription target annotation, the annotation ID, target protein, number of reference genes in the category, number of genes in the gene set and also in the category, expected number of genes in the category, ratio of enrichment, p-value from hypergeometric test and p-value adjusted for multiple testing. The gene symbols, Entrez IDs and Ensemble IDs are shown for each gene within an annotation. All information is adapted from WebGestalt (http://bioinfo.vanderbilt.edu/webgestalt; Zhang et al., 2005; Wang et al., 2013)*.

To investigate if this link to *Lef1* is common to many adult neurogenesis genes, or just our candidates, a list of genes expressed in Nestin-positive cell stages was retrieved from the MANGO database and was tested for enrichment in transcription factor targets using WebGestalt. This also revealed a significant enrichment for targets of *Lef1* (12 / 35 genes, adjP = 2.44 × 10^−8^).

**Figure 3:**
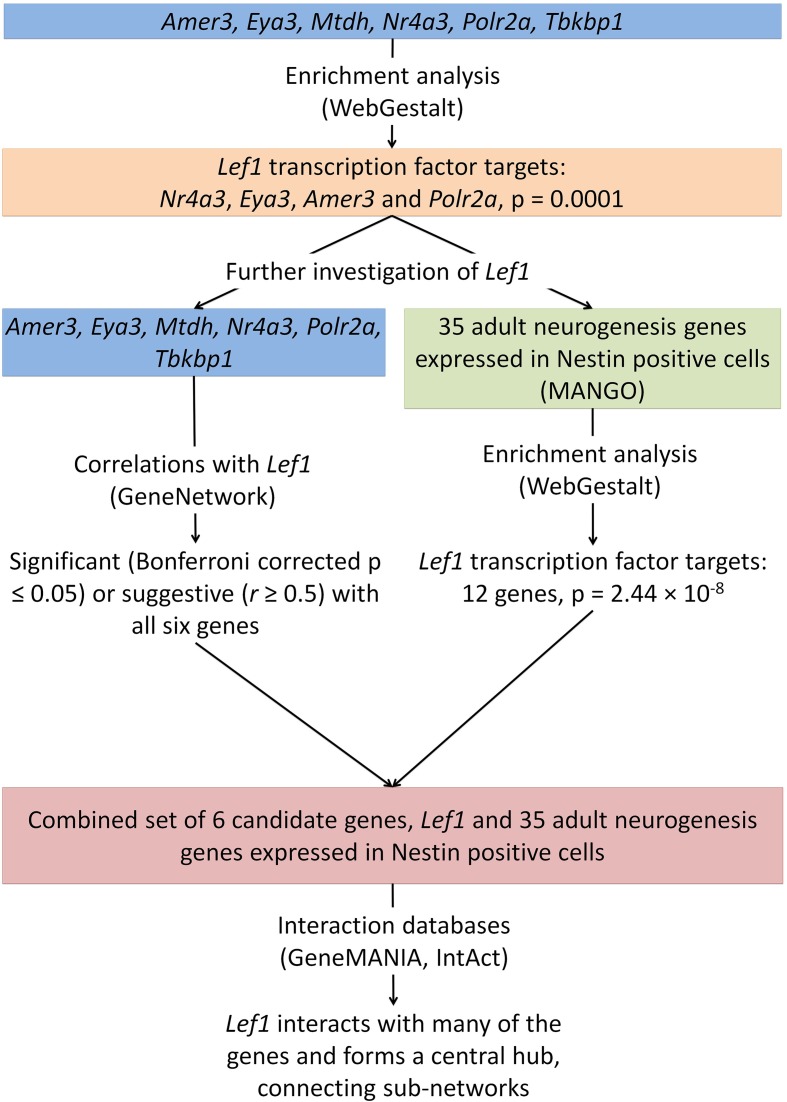
**The integration of data from several online tools identifies a novel role for Lef1 in adult hippocampal neurogenesis**. Enrichment analysis was carried out on the six candidate genes identified (Figure 1) and for targets of the transcription factor Lef1. This was further investigated using enrichment of genes known to be expressed in Nestin positive cells, correlation analysis between our candidate genes and *Lef1*, and the use of interaction databases to find links between our candidates.

These enrichment analyses suggest a possible novel role of *Lef1* as a key transcriptional regulator in proliferating neural precursor cells (Figure 3).

### Hippocampal expression patterns of lef1

To investigate if expression levels of *Lef1* correlate with expression of our candidate genes, correlation matrices were produced for the BXD and CXB strains. This shows suggestive correlations between our candidate genes and *Lef1* (Table 2). Although the correlation is generally greater (larger *r-*values) in the CXB set compared to the BXD, the significance is lower (larger *p*-values), due to far fewer lines being used (*n* = 71 vs. *n* = 15). However, in both sets, these correlations are not as strong as the correlations between our candidates and *Nes*. This suggests that *Lef1* is only one of several factors influencing the expression level of our genes of interest.

**Table 2:**
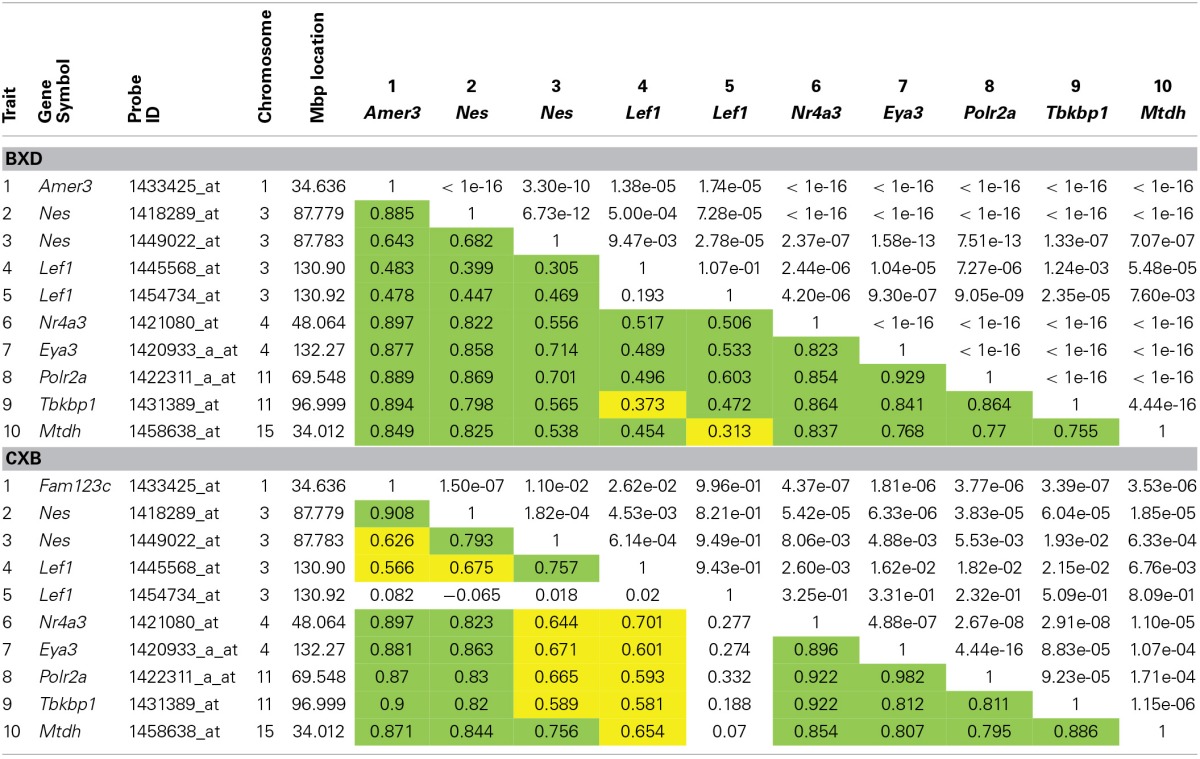
**Correlation matrices for hippocampal expression of our six candidate genes, *Nes* and *Lef1* in both BXD and CXB genetic reference populations**.

*On the left hand side of the matrix is the Pearson correlation value, and on the right hand side are the corresponding p-values. Statistically significant r-values (when Bonferroni corrected for the 45 comparisons being made; 0.05/45 = *p* < 0.0011) are colored green, with nominally significant values (p < 0.05) are colored yellow. Values are taken from GeneNetwork*.

The Allen Brain Map showed very little expression of *Lef1* in the hippocampus. However, only a small subset of hippocampal cells are proliferative, and *Lef1* may only be functioning at one stage in the cell cycle. To investigate whether a strong hippocampal *in situ* signal is a prerequisite for involvement in neurogenesis, a list of 35 genes from the MANGO database which are known to be expressed in Nestin-positive cells (Table 3) were examined in the Allen Mouse Brain Atlas for hippocampal expression. This showed that four genes known to be involved in adult hippocampal neurogenesis; *Id1* (Nam and Benezra, 2009), *Neurog2* (Ozen et al., 2007; Roybon et al., 2009), *Eomes* (Hodge et al., 2008; Azim et al., 2013) and *Kdr* (Cao et al., 2004; Warner-Schmidt and Duman, 2007; Segi-Nishida et al., 2008; Bernal and Peterson, 2011) all had low expression levels, similar to *Lef1*. Indeed Nestin itself appears in the Allen Brain Atlas to be poorly expressed, despite it being the dominant marker of the proliferating cell population. This indicates that although high transcript expression in the hippocampus might be supportive of a role in adult neurogenesis, it is certainly not necessary.

**Table 3:**
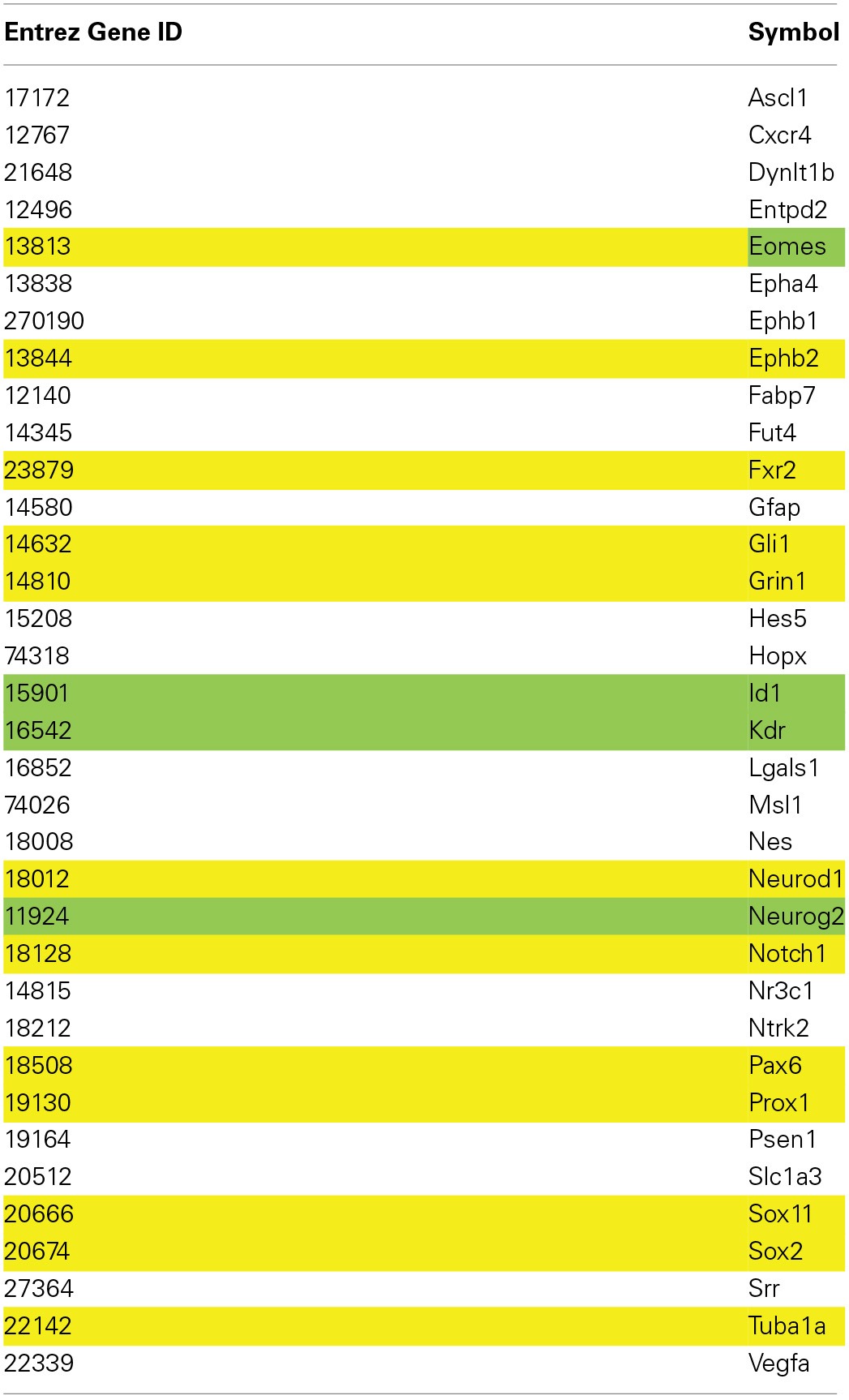
**List of MANGO genes expressed in Nestin positive cells**.

*Entrez gene IDs and symbols are shown for 35 genes which are known to be involved in adult neurogenesis and are expressed in NES positive cells. Those which are targeted by the Lef1 transcription factor are colored yellow (from WebGestalt). Those colored green have no hippocampal expression in the Allen mouse brain data (Lein et al., 2007; Allen Institute for Brain Science, 2014; http://www.brain-map.org)*.

### Predicted and known interactions with genes involved in adult neurogenesis

**Figure 4:**
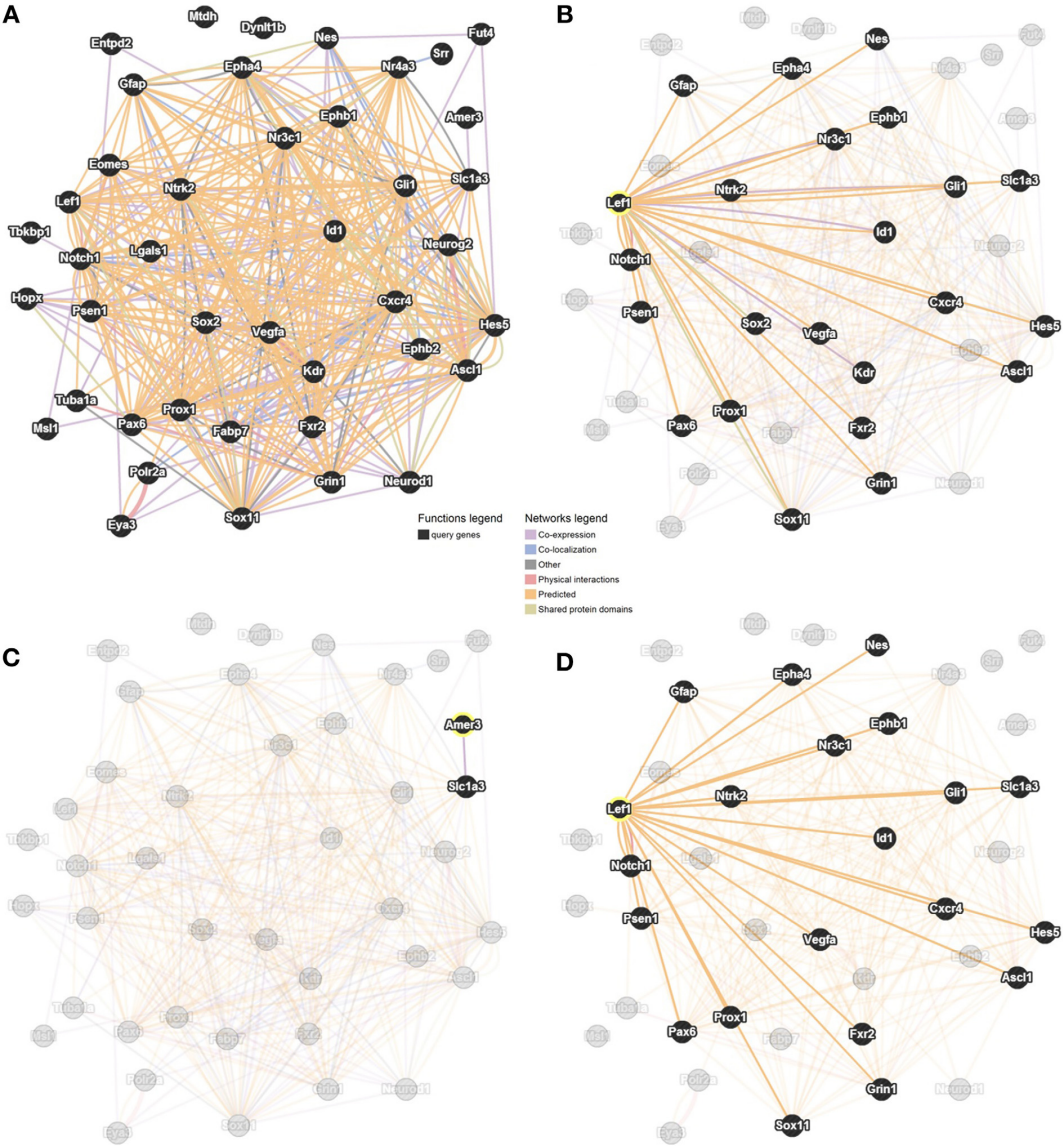
**Interaction network between the six candidate genes, *Lef1* and 35 MANGO genes expressed in Nestin-positive cells**. Adapted from GeneMANIA (http://genemania.org; Mostafavi et al., 2008; Warde-Farley et al., 2010) with links based on co-expression, co-localization, physical and genetic interactions, as well as shared protein domains. The network is shown with all genes and all interactions highlighted **(A**), only genes with a connection to *Lef1* highlighted **(B)**, only genes with a connection to Amer3 highlighted **(C)**, or highlighting restricted to genes with a physical, or predicted physical, interaction with *Lef1* **(D)**.

Our method, outlined above, allowed the identification of six novel candidate genes as being associated with adult hippocampal neurogenesis, and revealed that *Lef1* acts as a central hub between these genes. We employed GeneMANIA to predict interactions between the 35 MANGO genes expressed in Nestin positive cells, the six candidate genes and *Lef1*. This showed numerous interactions among all of the submitted genes (Figure 4A). In particular we saw a large number of interactions between *Lef1* and the 35 genes expressed in Nestin-positive cells (Figure 4B). In contrast, Amer3, which also appeared to be a good candidate, showed few interactions (Figure 4C), and none when only physical and predicted physical interactions are used whereas, when only considering the same interactions, the network surrounding Lef1 was essentially unchanged (Figure 4D).

**Figure 5:**
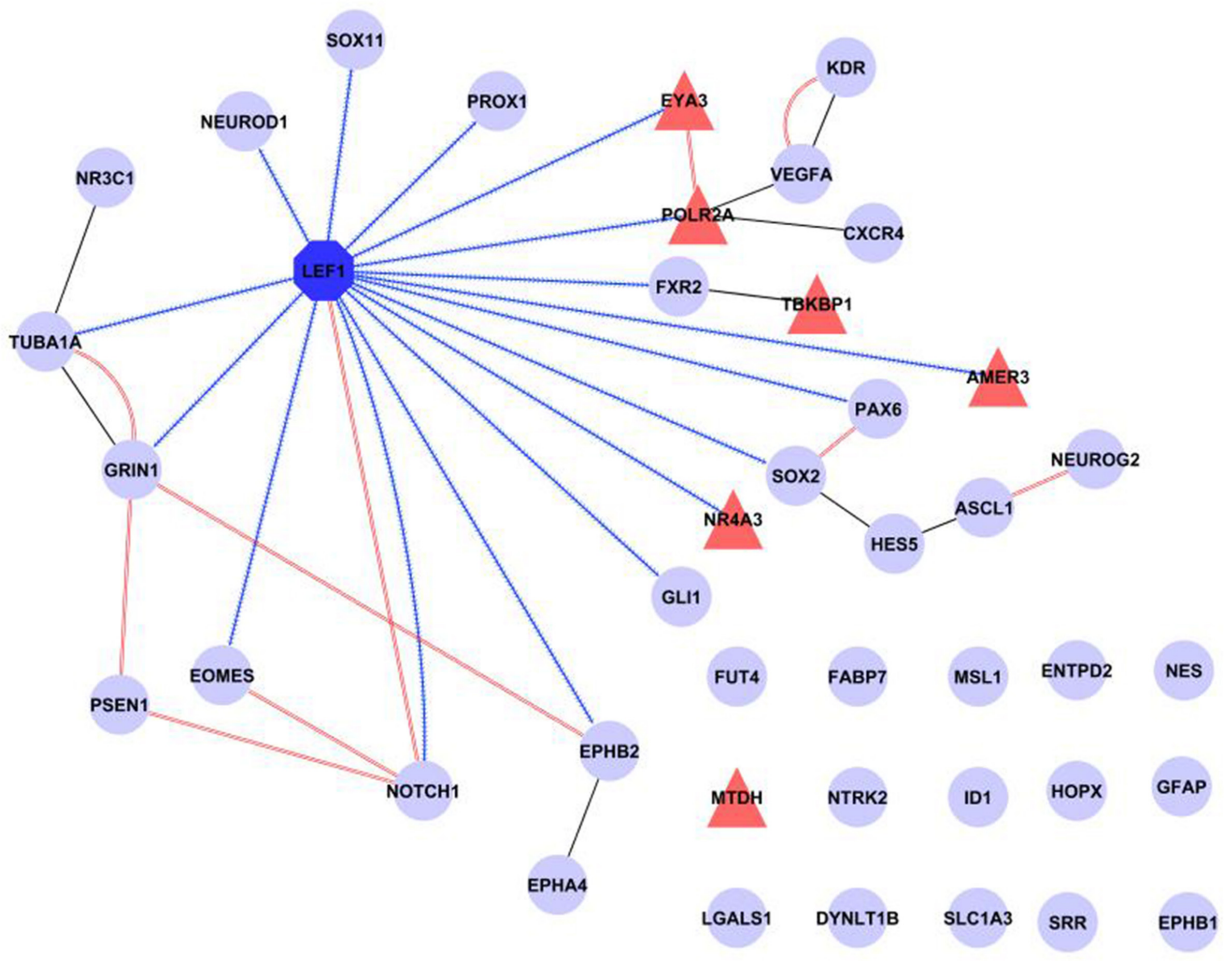
**Summary of known interactions shows a central role for Lef1**. The transcription factor Lef1 (dark blue octagon) is central in a network comprised of our six candidate genes (red triangles) and 35 genes expressed in Nestin positive cells (pale blue circles). Transcription factor targets (from WebGestalt) are shown as contiguous blue arrows, protein-protein interactions (from IntAct) as black lines and physical interactions (from GeneMANIA) as double red lines. Figure produced in Cytoscape.

Finally, a network was created incorporating all our evidence for Lef1 as a candidate regulator, including transcription factor targets from WebGestalt and physical interactions from GeneMANIA, as well as additional known protein-protein interactions from IntAct (Figure 5). This shows that Lef1 not only targets many of our genes, but that it connects subsets of physically interacting genes together.

Taken together, the results presented above support the hypothesis that *Lef1* plays a gene regulatory role in type-1 and type-2 Nestin-positive cells in the adult hippocampal dentate gyrus.

## Discussion

Adult neurogenesis occurs in several mammalian species, although the rate of precursor proliferation and new neuron production vary considerably between species (Kempermann, 2012). It has been widely studied in the mouse, where strain differences are present as well (Kempermann et al., 1997a, 2006; Hayes and Nowakowski, 2002; Clark et al., 2011; Poon and Goldowitz, 2014). Anatomical regions known to support adult neurogenesis are the subventricular zone and the subgranular zone of the hippocampal dentate gyrus, which is the focus of our study. Our understanding of neurogenesis regulatory networks and the functional interactions between genes expressed in this area and their protein products is limited.

Our approach consisted of finding genes that: (a) correlate strongly with *Nes* gene expression, a defining protein of hippocampal stem cells (Lagace et al., 2007), in two recombinant inbred mouse lines, BXD and CXB; and (b) have enhanced expression in the hippocampal dentate gyrus. In this way, we aimed to leverage existing, publicly available, *in silico* data to search for novel markers of the Nestin-positive precursor cell population.

The differential search option in the Allen Brain Science Institute's database allowed the identification of enhanced gene expression in a specific brain region. However, data for the subgranular zone, which is located in the immediate vicinity of the granule cell layer, and is only 2–3 cells thick, is not available at the present time. Therefore, the entire dentate gyrus was used as a proxy and compared to the whole brain gray matter. Because the subgranular zone (SGZ) is a thin band of cells located at the boundary of granule cell layer, small inaccuracies in boundary definition or spatial normalization could severely affect the analysis results. Therefore, we decided upon using the whole dentate gyrus as our best candidate for differential search.

*Amer3* has a well-defined enhanced expression in the granule cell layer of the hippocampus, and is a particularly good candidate since it has already been linked to embryonic neurogenesis (Comai et al., 2010). Furthermore, it is an Apc membrane recruitment protein, and Apc has already been linked to adult neurogenesis (Imura et al., 2010). Apc inhibits the Wnt signaling pathway via degradation of β-catenin, whereas Amer3 enhances the expression of a β-catenin (Brauburger et al., 2014), suggesting they have antagonistic roles in this important pathway within neurogenesis (Varela-Nallar and Inestrosa, 2013). Amer3 also binds to the Wnt pathway regulator Conductin/Axin2, and unlike Amer1 and Amer2 has been shown to be a positive regulator of the Wnt-β catenin signaling (Brauburger et al., 2014). This suggests that *Amer3* is indeed a good candidate for a novel molecular marker of this important cell population.

The reason that we found only a small set of genes using the differential search of Allen Brain Atlas resources is probably due to larger regions being used for our analysis (i.e., the whole hippocampus for *Nes* correlation and the dentate gyrus of the hippocampus for the differential gene expression search), as small but significant changes within the subgranular cell layer may be obscured by signals from other cell populations. Further refinements of the atlas and availability of a more spatially refined expression data set would likely yield a greater overlap between these two approaches.

Our protein-protein interaction and enrichment analysis revealed that four out of our six candidate genes are targeted by the Lef1 transcription factor, and the remaining two have close relations to it. Lef1 is a downstream effector of the Wnt/β-catenin pathway (Mazumdar et al., 2010), which is important in adult neurogenesis (Machon et al., 2003; Lie et al., 2005; Kuwabara et al., 2009; Wexler et al., 2009; Varela-Nallar and Inestrosa, 2013; Wisniewska, 2013). *Lef1* is expressed in cultured hippocampal neural stem cells in response to activation of the Wnt signaling pathway (Cui et al., 2011). Our evidence and the literature both suggest that genes known to be involved in hippocampal adult neurogenesis are targets of Lef1, an important factor in generating granule cells in the dentate gyrus during development (Galceran et al., 2000). The only two genes not targeted by Lef1 can be closely associated with it: Mtdh regulates the expression of *Lef1* (Hu et al., 2009; Yoo et al., 2009), and Tbkbp1 physically interacts with a known adult neurogenesis proteins Fxr2, which is expressed in Nestin positive cells and is a Lef1 target (Figure 5).

Combined with the established role of the Wnt signaling pathway in adult hippocampal neurogenesis (Lie et al., 2005) together with the known association between *Lef1* and β-catenin (Behrens et al., 1996), our results here suggest that *Lef1* is an important part of the Wnt-controlled regulation of neural precursor function in the adult dentate gyrus.

While our approach has limitations, such as relying on one single marker of proliferation, and on spatial proxies for the SGZ, we believe that it proposes a novel method. By repeating a similar procedure, as we followed using Nestin, and then imposing spatial constraints for other markers associated with neurogenesis (e.g., *Sox2, Prox1, NeuN*, or *Dcx*), one might expect to produce larger sets of gene candidates and better understand their roles in the various stages of neurogenesis. It is our hope that this can lead to a better understanding of adult neurogenesis and its relationship with its developmental counterpart, as well as give additional insight into the functional relevance of a process that has been demonstrated in several mammalian species (Amrein et al., 2011; Kempermann, 2012; Patzke et al., 2013).

In this study we demonstrate a strategy for finding novel candidate genes linked to adult neurogenesis in the murine hippocampus, using data available in the public domain. By integrating data from several public resources, this method presents an avenue for generating novel hypotheses *in silico*, and potential gene networks which can be tested in the future *in vitro* or *in vivo*.

## Conflict of interest statement

The authors declare that the research was conducted in the absence of any commercial or financial relationships that could be construed as a potential conflict of interest.
